# The relationship between self-compassion and non-suicidal self-injury among Chinese college students: the chain mediating roles of psychological resilience and well-being

**DOI:** 10.3389/fpsyg.2026.1820990

**Published:** 2026-05-12

**Authors:** Gang Hu, Wei Feng, Fupeng Sheng, Xinxin Qin

**Affiliations:** 1 Mental Health Education and Guidance Center, Jiangsu University, Zhenjiang, China; 2 Mental Health Center, Jiangsu Food and Pharmaceutical Science College, Huaian, China; 3 Student Affairs Department, Jiangsu University, Zhenjiang, China; 4 Teachers College (School of Education), Jiangsu University, Zhenjiang, China; 5 Institute of High-Quality Development of Basic Education at Jiangsu University, Zhenjiang, China

**Keywords:** self-compassion, psychological resilience, well-being, non-suicidal self-injury, chain mediating role

## Abstract

**Objectives:**

This study aimed to explore the chain mediating roles of psychological resilience and well-being between self-compassion and non-suicidal self-injury (NSSI) among Chinese college students from the perspective of positive psychology.

**Methods:**

A total of 1,083 college students were recruited from three universities in Zhenjiang, Nanchang, and Fuzhou, China. Participants voluntarily completed the Self-Compassion Scale, Connor–Davidson Resilience Scale, Index of Well-Being Scale, and Adolescents Self-Harm Scale.

**Results:**

The results showed that: (1) Self-compassion and its components, psychological resilience, and well-being and its dimensions were all significantly positively correlated with each other, and all were significantly negatively correlated with NSSI. (2) Well-being mediated the relationship between self-compassion and NSSI, while psychological resilience and well-being sequentially mediated the association between self-compassion and NSSI. (3) The indirect association pathways between the components of self-compassion and NSSI might differ: the negative association between common humanity and NSSI could be manifested through the independent mediating role of well-being as well as the chain mediating roles of psychological resilience and well-being; whereas self-kindness and mindfulness were negatively associated with NSSI only through the chain mediating roles of psychological resilience and well-being. (4) The indirect effect of mindfulness on NSSI was significantly greater than that of the other components of self-compassion.

**Conclusion:**

Psychological resilience and well-being are important mediating variables in the relationship between self-compassion and NSSI among college students. Specifically, mindfulness may serve as a key component enabling self-compassion to exert its protective effects, and well-being may play a more crucial mediating role between self-compassion and NSSI.

## 1 Introduction

Non-suicidal self-injury (NSSI) refers to the deliberate, direct damage to one’s own body tissue without suicidal intent, including behaviors such as cutting, scratching, burning, scraping, and tearing (Moloney et al., 2024). A study showed that the prevalence of NSSI among Chinese college students ranged from 7.80% to 23.23% and was on an upward trend (Gao et al., 2025). NSSI poses severe threats to college students’ physical and mental health and social adjustment. It not only contributes to psychological disorders and behavioral problems but also increases the risk of suicide. Against this background, it is of great significance to explore the influencing factors and underlying mechanisms of NSSI among college students.

Although numerous studies have examined the antecedents and consequences of NSSI from various perspectives, most have focused on risk factors that trigger and maintain NSSI, with relatively less attention paid to protective factors. With the development of positive psychology, the beneficial effects of self-compassion on individuals’ problematic behaviors have received increasing attention. However, few previous studies have systematically investigated the relationship between self-compassion and NSSI among college students. Based on the current research status, the present study recruited Chinese college students as participants to examine the association between self-compassion and NSSI, with the aim of promoting scientific prevention and effective intervention for NSSI.

## 2 Research hypotheses

### 2.1 Self-compassion and NSSI

Self-compassion is defined as individuals’ ability to treat themselves with tolerance and understanding, and to maintain kindness and care toward themselves when facing setbacks, misfortunes, and difficulties (Neff, 2003b). According to General Strain Theory (GST) and the Experiential Avoidance Model (EAM) of self-injury, individuals exposed to stressful events may tend to develop negative emotions such as anxiety, depression, and anger. Driven by these emotions, they may adopt inappropriate coping strategies including aggression toward others or self-harm to relieve stress or express distress (Agnew, 1992; Chapman et al., 2006; Liu et al., 2022).

As an adaptive emotion-regulation strategy, self-compassion can effectively improve individuals’ own satisfaction of basic psychological needs (Miyagawa et al., 2023), enhance psychological stability (Zhao et al., 2025), and provide them with crucial self-regulatory resources, thereby encouraging them to adopt healthy behaviors instead of negative coping patterns such as self-injury (Terry and Leary, 2011). Empirical studies have also shown that self-compassion helps individuals reduce negative emotions and lower the risk of NSSI (Lichvárová et al., 2025; Neff and Germer, 2013).

In addition, previous studies have mostly focused on the assessment of overall self-compassion, with relatively little exploration of the effects of its components on individuals’ psychology and behavior. In fact, different components of self-compassion may exert distinct influences on the physical and mental health of individuals at different life stages. Based on the above considerations, the present study proposes Hypothesis H1: overall self-compassion and its components are negatively associated with NSSI among college students.

### 2.2 The mediating role of psychological resilience

Psychological resilience, also referred to as mental resilience or resilience, refers to individuals’ ability to effectively cope with, recover rapidly from, and maintain a healthy psychological state in the face of loss, adversity, threat, or other major stressful situations (Garmezy, 1991). According to the Experiential Avoidance Model (EAM), individuals experiencing negative emotional distress may adopt maladaptive behaviors such as NSSI behaviors for relief. Although this approach may provide temporary emotional comfort, it continuously strengthens the association between NSSI and negative emotions (Chapman et al., 2006).

In contrast, individuals with higher levels of psychological resilience exhibit better adaptive capacity, showing active adjustment and calm coping when facing stress, and perceiving less psychological distress (Sun et al., 2025), thereby reducing the likelihood of NSSI. Previous studies have indicated that psychological resilience, as a protective factor enabling individuals to effectively cope with adversity, plays a positive role in promoting mental health and reducing NSSI behaviors (Yuan et al., 2024).

How is psychological resilience formed and enhanced? From the perspective of resilience resource development, its formation and progression rely on internal factors such as cognition, emotion, and spirituality, and are gradually constructed through a series of psycho-behavioral processes (Zhu et al., 2019). When individuals face difficulties, self-compassion prompts them to employ adaptive emotion regulation strategies such as cognitive reappraisal and acceptance, while reducing maladaptive strategies like expressive suppression. This further facilitates the development and accumulation of positive physical and psychological resources, providing an essential foundation for the growth of psychological resilience.

Some empirical studies have found that self-compassion is not only significantly positively correlated with psychological resilience but also effectively predicts changes in psychological resilience (Ime et al., 2025; Kemper et al., 2015). Based on these findings, the present study proposes Hypothesis H2: psychological resilience mediates the relationship between overall self-compassion (and its components) and NSSI among college students.

### 2.3 The mediating role of well-being

Well-being is defined as a comprehensive cognitive and emotional evaluation that arises when individuals conduct an overall assessment of their quality of life based on internally established criteria (Diener et al., 2003). As a key indicator for evaluating the positivity of individuals’ psychological development and their mental health status, well-being can intuitively reflect individuals’ adaptive conditions in terms of emotions, cognition, social functions, and other aspects, exerting multi-dimensional and sustained beneficial effects on the maintenance of individuals’ physical and mental health and the improvement of their social adaptability. Declined well-being indicates weakened positive mental health, making individuals more prone to negative emotional cycles and more likely to adopt NSSI as a coping strategy to distract from emotions or relieve distress when confronted with pressure and pain. Existing empirical studies support this inference. For example, a survey of American adolescents found that those with lower levels of well-being were more likely to engage in NSSI behaviors (Burke et al., 2018). This raises the critical question: what factors can effectively promote individual well-being?

The Sustainable Happiness Model (SHM) clearly indicates that individual well-being is comprehensively influenced by three factors: heredity, environment, and intentional activities. Compared with stable and innate genetic factors, as well as dynamic and complex environmental factors constrained by external conditions, intentional activities help individuals proactively adjust their mentality and behaviors to enhance well-being, which is particularly more practically significant for individuals (Lyubomirsky et al., 2005). Self-compassion emphasizes accepting one’s own deficiencies with self-kindness, acknowledging negative experiences with mindfulness, and reinterpreting failures and setbacks from a common humanity perspective. It promotes emotional transformation and stress coping through a series of active regulatory strategies and essentially constitutes an intentional activity that may contribute to improving individual well-being. This has been confirmed in previous relevant studies (Neff and Germer, 2013; Tan, 2019). Based on these findings, the present study proposes Hypothesis H3: well-being mediates the relationship between overall self-compassion (and its components) and NSSI among college students.

### 2.4 The chain mediating roles of psychological resilience and well-being

Based on the above analyses, it can be inferred that psychological resilience and well-being may be important mediating variables between self-compassion and NSSI, but what is the relationship between them? According to the conservation of resources (COR) theory, the maintenance and construction of resources provide a practical pathway for individuals to pursue well-being, whereas the depletion and loss of resources run counter to this (Halbesleben et al., 2014). Psychological resilience, as an inherent positive psychological resource, can help individuals build additional positive psychological resources, thereby achieving gains in new psychological resources and personal growth.

This process of resource accumulation and development contributes to maintaining a sound physical and mental state, effectively buffers the depletion of psychological resources caused by stress, reduces negative emotions and maladjustment problems, and thus sustains and enhances well-being. Empirical studies have also found that there is a significant positive correlation between psychological resilience and well-being (Ni et al., 2025), and psychological resilience has a certain predictive effect on well-being (Mao et al., 2024). Based on these findings, the present study proposes Hypothesis H4: psychological resilience and well-being play a chain mediating role in the relationship between overall self-compassion (and its components) and NSSI among college students.

**Figure 1:**
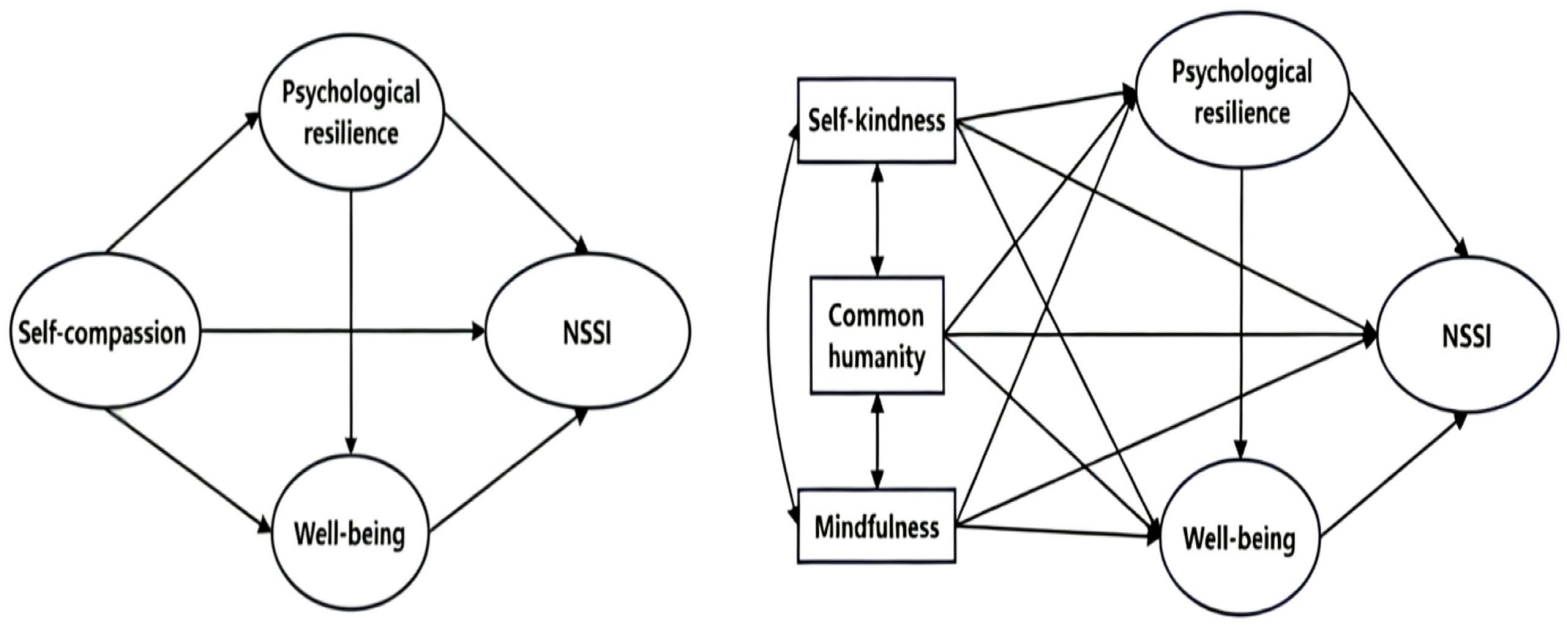
Diagram of the hypothetical model.

Based on the existing theoretical and empirical studies, this study recruited college students from Chinese universities as participants and constructed a hypothetical model of the relationships among self-compassion, psychological resilience, well-being, and NSSI, as shown in Figure 1.

## 3 Methods

### 3.1 Study design

This study employed a cross-sectional quantitative design and complied with the ethical principles of the Declaration of Helsinki. A convenience sampling method was adopted to recruit undergraduate students from three universities as research participants, and a questionnaire survey was administered for data collection.

This sampling method was selected for three main reasons. First, given that NSSI involves personal privacy and mental health issues, convenience sampling helps establish trust with participants under anonymity and confidentiality, thereby improving data authenticity. Second, this study focuses on hypothesis testing of variable relationships and theoretical model verification rather than estimation of population parameters, for which convenience sampling is sufficiently effective and appropriate. Third, college students are a high-priority and high-risk group in NSSI research, and the established cooperation with partner universities ensures standardized, efficient, and high-quality data collection.

### 3.2 Study setting

This study was conducted from September 1 to October 31, 2025, at three full-time regular undergraduate universities in Zhenjiang, Nanchang, and Fuzhou, with full-time undergraduate students as participants. All three institutions are local comprehensive universities with a large undergraduate enrollment and a comprehensive array of academic disciplines. Given that the study focuses on issues related to NSSI, the specific names of the universities are withheld to safeguard participants’ privacy and prevent potential adverse effects on the institutions.

Prior to data collection, formal ethics approval was obtained for this study, along with necessary permission and support from the relevant administrative departments, colleges, and counselors of each participating university. Data were collected in intact classes during free periods within the academic teaching week. The questionnaire surveys were administered in multimedia classrooms or study rooms on campus, which were quiet, well-lit, and adequately ventilated to ensure that participants could complete the questionnaires independently and steadily.

### 3.3 Participants

This study recruited undergraduate students from Grade 1 to Grade 4 at three full-time regular universities as participants. During the instructional weeks, counselors and college administrators selected intact classes, posted recruitment announcements through class online messaging groups (e.g., WeChat and QQ), and recruited eligible students on a voluntary basis. Paper-and-pencil questionnaires were administered to all participants in a unified group setting.

The inclusion criteria were as follows: (1) being officially registered full-time undergraduate students; (2) having normal auditory perception; (3) possessing normal cognitive and language comprehension abilities to complete the questionnaire independently; (4) voluntarily participating and providing written informed consent.

The exclusion criteria were as follows: (1) being in the acute episode stage of severe psychological or mental disorders (identified by counselors or self-reported by students); (2) having severe cognitive impairment that prevents understanding of questionnaire items; (3) participating in other similar research projects concurrently; (4) submitting invalid questionnaires due to patterned responses, excessive missing data, or other comparable issues.

### 3.4 Measures

#### 3.4.1 Self-Compassion Scale

The Self-Compassion Scale (SCS) was originally developed by Neff (2003a). In this study, the Chinese version of the SCS, revised by Gong et al. (2014), was used to measure self-compassion among college students. The scale consists of 12 items with three dimensions, namely, self-kindness, common humanity, and mindfulness, and is assessed on a 5-point scale ranging from 1 (never) to 5 (always). The total theoretical score ranges from 12 to 60, with higher scores indicating a higher level of self-compassion. A sample item includes “When feeling depressed, I try to regulate my emotions with a positive and open-minded attitude.” Previous studies have indicated that the scale is applicable to Chinese college students and exhibits satisfactory reliability and validity (Yang et al., 2021). The original scale had a Cronbach’s *α* coefficient of 0.770 (Gong et al., 2014). In the present study, the Cronbach’s *α* coefficient for the scale was 0.805.

#### 3.4.2 Connor–Davidson Resilience Scale

The Connor–Davidson Resilience Scale (CD-RISC) was originally developed by Connor and Davidson (2003). The 10-item Connor–Davidson Resilience Scale (CD-RISC-10) revised by Wang et al. (2010) was used to assess psychological resilience among college students. This unidimensional scale consists of 10 items. Each item is rated on a 5-point Likert scale ranging from 1 (never) to 5 (always). The total theoretical score ranges from 10 to 50, with higher scores indicating a stronger level of psychological resilience. A sample item includes “When facing difficulties, I try to see the positive side of things.” The original scale had a Cronbach’s *α* coefficient of 0.910 (Wang et al., 2010). In the current study, the scale demonstrated excellent internal consistency, with a Cronbach’s *α* coefficient of 0.915.

#### 3.4.3 Index of Well-Being Scale

The Index of Well-Being Scale (IWB) developed by Campbell (1976) and revised by Wang et al. (1999) was used to assess individuals’ level of well-being. The scale consists of two components: the Index of General Affect Scale (8 items) and the Life Satisfaction Scale (1 item), with a total of 9 items. Each item of the scale is rated on a 7-point Likert scale, ranging from 1 (strongly disagree) to 7 (strongly agree). The total score is calculated as the weighted sum of the mean score of the Overall Affective Index Scale (weight = 1) and the score of the Life Satisfaction Scale (weight = 1.1). The total theoretical score ranges from 2.1 (least happy) to 14.7 (most happy). Higher scores indicate a higher level of well-being experienced by individuals. Some examples of the scale include “bored-interested” and “hopeless-hopeful.” The scale has been validated for reliability and validity across diverse cultural backgrounds, confirming its good applicability in both Eastern and Western populations (Chen et al., 2019). The test–retest reliability of the original scale was 0.849 (Wang et al., 1999). The scale demonstrated excellent reliability in the current study, with a Cronbach’s *α* coefficient of 0.910.

#### 3.4.4 Adolescents Self-Harm Scale

The Adolescents Self-Harm Scale (ASHS), originally developed by Zheng (2006) and revised by Feng (2008), was used to assess NSSI behaviors among participants. The scale consists of 18 items, and each item comprises two parallel paired parts: the frequency of NSSI behavior and the severity of NSSI behavior. The frequency of NSSI behavior is rated on a 4-point scale: 0 times, 1 time, 2–4 times, and 5 times or more, scored 0 to 3, respectively. The severity of NSSI behavior is rated on a 5-point scale: none, mild, moderate, severe, and extremely severe, scored 0 to 4, respectively. The score for each item is calculated by multiplying the frequency score by the severity score, and the total score of NSSI is obtained by summing the scores of all items. The total theoretical score ranges from 0 to 216, with higher total scores indicating more severe NSSI behaviors. A sample item is: “Intentionally scratching one’s skin with glass, a knife, or other sharp objects.” Previous studies have indicated that the scale is applicable to Chinese college students and exhibits satisfactory reliability and validity (Chen et al., 2020). The original scale had a Cronbach’s *α* coefficient of 0.850 (Feng, 2008). In this study, the scale showed good reliability with a Cronbach’s *α* coefficient of 0.858.

### 3.5 Data collection

Prior to formal implementation, this study was reviewed and approved by the Medical Ethics Committee of Jiangsu University (Approval No. JSDX20250701020), and was conducted in strict accordance with the ethical principles of the Declaration of Helsinki. Data collection was carried out between September 1 and October 31, 2025, using offline paper-and-pencil questionnaires administered by trained postgraduate students majoring in psychology. Before formal assessment, the four scales were assembled in random order. After obtaining informed consent from school and college administrators, counselors, and the students themselves, participants were informed of their anonymity, their right to withdraw from the study at any time, and relevant procedural instructions. Group testing was conducted on a class basis. The standardized instructions were read aloud by the trained experimenters, and participants completed the questionnaires independently and conscientiously in accordance with the instructions and requirements. All questionnaires were collected immediately and uniformly on the spot upon completion.

### 3.6 Statistical analysis

All statistical analyses were performed using SPSS 25.0 and Mplus 8.3. SPSS 25.0 was used to test for the presence of common method bias, descriptive statistics, and correlation analyses. Mplus 8.3 was used to perform confirmatory factor analysis (CFA) and construct structural equation models to examine the relationship between self-compassion and NSSI among college students, together with the chain mediating roles of psychological resilience and well-being.

## 4 Results

### 4.1 Sample characteristics

A total of 1,200 questionnaires were distributed, and 1,083 valid questionnaires were obtained, resulting in an effective rate of 90.25%. Among the participants, 569 were male (52.54%) and 514 were female (47.46%). In terms of grade level, 656 were freshmen (60.57%), 129 were sophomores (11.91%), 221 were juniors (20.41%), and 77 were seniors (7.11%). The age of the participants ranged from 17 to 29 years old, with a mean age of 19.55 ± 1.69 years.

### 4.2 Reliability and validity analysis of measurement tools

Prior to examining the relationships among variables, the present study first evaluated the reliability and validity of all scales to ensure the quality and effectiveness of the measurement data. For reliability assessment, both Cronbach’s *α* and McDonald’s *ω* coefficients were calculated, with the results presented in Table 1. The findings demonstrated that Cronbach’s *α* coefficients for all variables were above 0.80, and McDonald’s *ω* coefficients for all scales also exceeded 0.80, which were higher than the cutoff value of 0.70 and met psychometric standards. These results indicated that all scales possessed satisfactory internal consistency and were suitable for subsequent statistical analyses.

**Table 1:** Reliability and validity of the scales.

Variables	Cronbach’s *α*	McDonald’s *ω*	*χ*^2^/*df*	CFI	TLI	RMSEA	SRMR
Self-compassion	0.805	0.813	6.006	0.930	0.909	0.068	0.050
Psychological resilience	0.915	0.917	8.862	0.952	0.938	0.085	0.031
Well-being	0.910	0.914	10.737	0.953	0.935	0.095	0.032
Non-suicidal self-injury	0.858	0.887	—	—	—	—	—

For validity assessment, confirmatory factor analysis (CFA) was performed to examine the structural validity of the Self-Compassion Scale, Connor–Davidson Resilience Scale, and Index of Well-Being Scale, with the corresponding results displayed in Table 1. The fit indices of the three measurement models were all within acceptable ranges, suggesting that these scales demonstrated adequate structural validity. Regarding the validity of the Adolescents Self-Harm Scale, this study provided support from multiple sources of evidence. Previous studies have supported its satisfactory structural validity, discriminant validity, convergent validity, and criterion-related validity (Chen et al., 2020; Feng, 2008). In addition, before formal administration, experts in relevant fields were invited to assess the items of the scale, and they unanimously confirmed its acceptable content validity.

Notably, due to the highly skewed distribution and extremely high proportion of zero responses for self-injurious behavior, this variable was treated as an observed variable rather than a latent variable in the structural model. Furthermore, as the data failed to meet the basic assumptions of conventional CFA (e.g., normality and continuity), CFA was not conducted for this scale (Flora et al., 2012; Liang and Yang, 2016). Instead, its psychometric properties were comprehensively supported by internal consistency reliability indices combined with validity evidence from prior studies.

### 4.3 Common method bias test

Since all data in this study were collected via self-report measures, the resulting data were potentially subject to common method bias. To mitigate the adverse effects of common method bias on the results, procedural remedies were implemented during the questionnaire administration stage in accordance with established recommendations (Zhou and Long, 2004). Specifically, participants were informed that all data would be used exclusively for academic research and kept strictly confidential, and responses were collected anonymously. Additional procedural controls included separating different scales, using distinct rating formats, incorporating reverse-coded items within some scales, and providing all participants with standardized instructions.

Following data collection, Harman’s single-factor test was conducted on all items of the study variables to assess the presence of common method bias. The results indicated that there were 9 factors with eigenvalues greater than 1, and the first factor accounted for only 25.29% of the total variance. This value fell within the acceptable range, as it was below the critical threshold of 40% (Zhou and Long, 2004).

The confirmatory factor analysis (CFA) version of Harman’s single-factor test was further conducted to evaluate the severity of common method bias. When all indicators were loaded onto a single common factor, the model fit indices were as follows: *χ^2^/df* = 13.606, CFI = 0.478, TLI = 0.455, RMSEA = 0.108, SRMR = 0.125, all of which fell well below the standard thresholds for acceptable model fit, implying that a single latent factor did not account for the bulk of the covariance among the measures.

As a result, this finding suggested that common method bias was unlikely to be a substantial issue in this study, and the data were suitable for subsequent empirical analyses.

### 4.4 Descriptive statistics and correlation analysis

Table 2 displays the descriptive statistics and correlations of the study variables. The results of the correlation analyses showed that self-compassion and its components, psychological resilience, as well as well-being and its dimensions were all significantly positively correlated with each other. In contrast, self-compassion and its components, psychological resilience, and well-being and its dimensions were all significantly negatively correlated with NSSI.

**Table 2:** Descriptive statistics and correlations among variables.

Variables	M	SD	1	2	3	4	5	6	7	8
1. Self-kindness	11.609	2.116	1							
2. Common humanity	13.335	3.223	0.409^***^	1						
3. Mindfulness	19.925	3.267	0.485^***^	0.276^***^	1					
4. Self-compassion	44.869	6.606	0.760^***^	0.755^***^	0.785^***^	1				
5. Psychological resilience	38.947	6.189	0.492^***^	0.349^***^	0.713^***^	0.680^***^	1			
6. Overall affective	5.268	1.023	0.404^***^	0.384^***^	0.511^***^	0.570^***^	0.642^***^	1		
7. Life satisfaction	5.961	1.233	0.375^***^	0.340^***^	0.426^***^	0.497^***^	0.528^***^	0.695^***^	1	
8. Well-being	11.229	2.079	0.421^***^	0.391^***^	0.504^***^	0.575^***^	0.629^***^	0.904^***^	0.935^***^	1
9. Non-suicidal self-injury	0.905	3.842	−0.163^***^	−0.134^***^	−0.141^***^	−0.187^***^	−0.160^***^	−0.213^***^	−0.168^***^	0.205^***^

*p < 0.05, **p < 0.01, ***p < 0.001.

### 4.5 Analysis of the chain mediating effects of psychological resilience and well-being in the relationship between self-compassion and NSSI

To investigate the chain mediating effects of psychological resilience and well-being in the relationship between self-compassion and NSSI, structural equation modeling was conducted using Mplus 8.3. Given the relatively large number of items in the unidimensional scales for psychological resilience and NSSI employed in the present study, the factorial algorithm within item parceling strategies was applied to construct three indicators for each of the two scales, following recommendations from previous studies (Wu and Wen, 2011).

Prior to conducting structural equation modeling, the measurement model was evaluated first, and the results indicated that its goodness-of-fit indices were satisfactory: *χ^2^/df* = 3.908, CFI = 0.989, TLI = 0.980, RMSEA = 0.052, SRMR = 0.028. Consistent with the research hypotheses, the structural equation modeling was then established, and the validation results indicated that the goodness-of-fit indices of this model were satisfactory:*χ^2^/df* = 5.088, CFI = 0.977, TLI = 0.966, RMSEA = 0.061, SRMR = 0.039.

**Figure 2:**
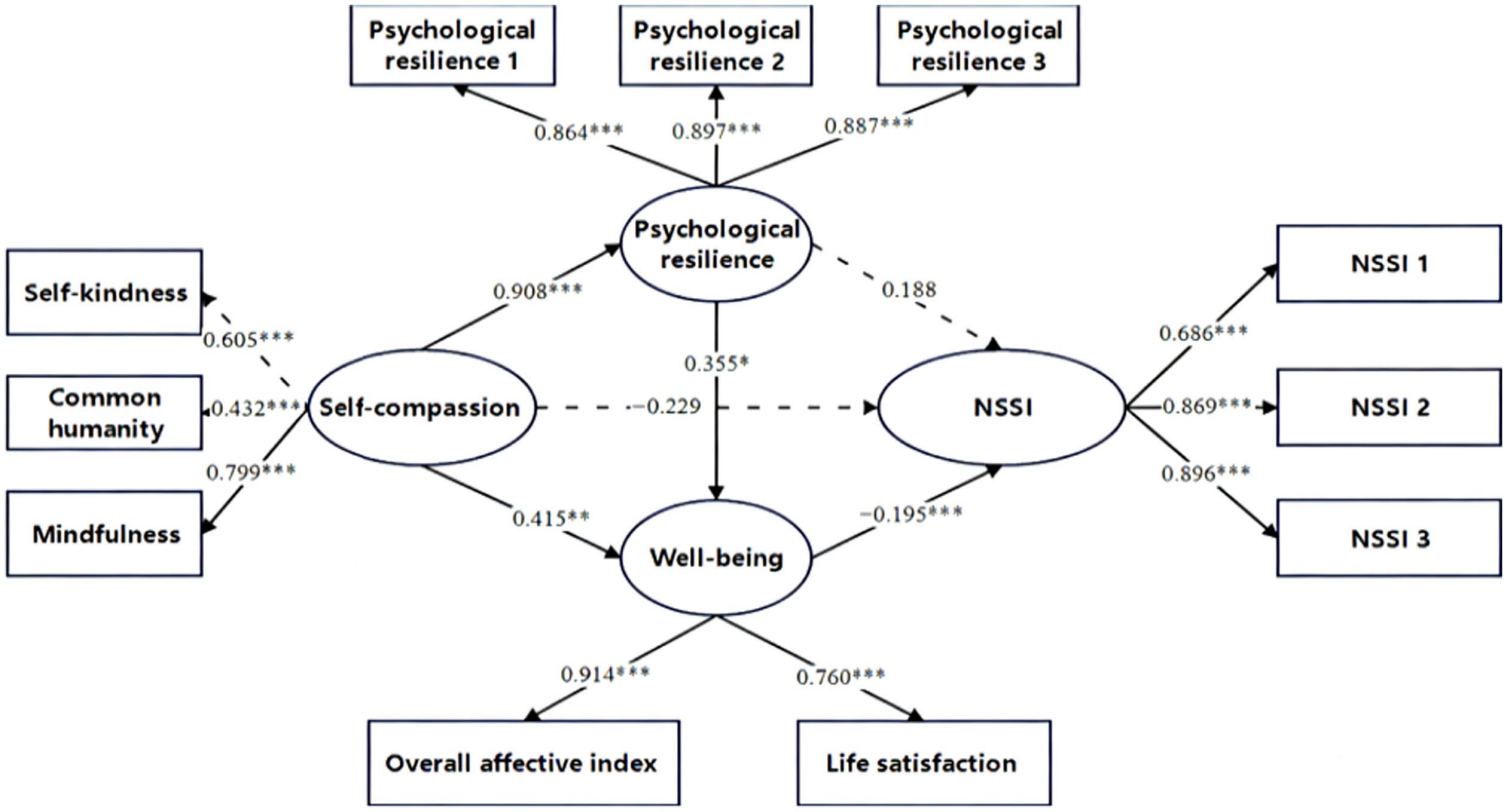
Diagram of the chain mediating effect model between overall self-compassion and NSSI. *p < 0.05, **p < 0.01, ***p < 0.001.

The results revealed that all direct paths in the model were statistically significant, with the exception of the direct effect of self-compassion on NSSI (*β* = −0.229, *t* = −1.409, *p* > 0.05) and the direct predictive path from psychological resilience to NSSI (*β* = 0.188, *t* = 1.300, *p* > 0.05). Analyses of the standardized path coefficients indicated that the influence of self-compassion on NSSI was significantly exerted through two distinct indirect pathways: one from self-compassion to NSSI via well-being, and the other from self-compassion to NSSI via psychological resilience and well-being in series. The standardized regression coefficients of each path in the model are presented in detail in Figure 2.

Bootstrap test was used to repeat the sampling 5,000 times to examine the significance of the multiple mediating effects in the model. As presented in Table 3, the 95% Bootstrap confidence intervals for both pathways did not include 0, indicating that both mediating pathways were statistically significant.

**Table 3:** Bootstrap analysis for the significance test of mediating effects.

Mediating model pathway	Estimated effect	95% CI	
		Lower	Upper
Self-compassion → psychological resilience → NSSI	0.171	−0.050	0.471
Self-compassion → well-being → NSSI	−0.081	−0.154	−0.031
Self-compassion → psychological resilience → well-being → NSSI	−0.063	−0.125	−0.005

### 4.6 Analysis of the chain mediating effects of psychological resilience and well-being in the relationship between components of self-compassion and NSSI

Prior to conducting structural equation modeling, the measurement model was evaluated first, and the results indicated that its goodness-of-fit indices were satisfactory: *χ^2^/df* = 4.921, CFI = 0.986, TLI = 0.971, RMSEA = 0.060, SRMR = 0.030. Structural equation modeling was performed with the components of self-compassion as independent variables, psychological resilience and well-being as mediating variables, and NSSI as the dependent variable. The overall model fit indices were satisfactory: *χ^2^/df* = 2.481, CFI = 0.992, TLI = 0.988, RMSEA = 0.037, SRMR = 0.028.

The results of the standardized path coefficients revealed that self-kindness, common humanity, and mindfulness did not significantly predict NSSI (*β* = 0.075, *t* = −1.597, *p* > 0.05; *β* = −0.015, *t* = −0.396, *p* > 0.05; *β* = −0.011, *t* = −0.211, *p* > 0.05), whereas they all exerted significant positive predictive effects on psychological resilience (*β* = 0.156, *t* = 5.249, *p* < 0.001; *β* = 0.127, *t* = 4.944, *p* < 0.001; *β* = 0.635, *t* = 25.168, *p* < 0.001). In addition, common humanity significantly and positively predicted well-being (*β* = 0.171, *t* = 6.016, *p* < 0.001), while self-kindness and mindfulness showed no significant predictive effects on well-being (*β* = 0.050, *t* = 1.548, *p* > 0.05; *β* = 0.018, *t* = 0.443, *p* > 0.05).

**Figure 3:**
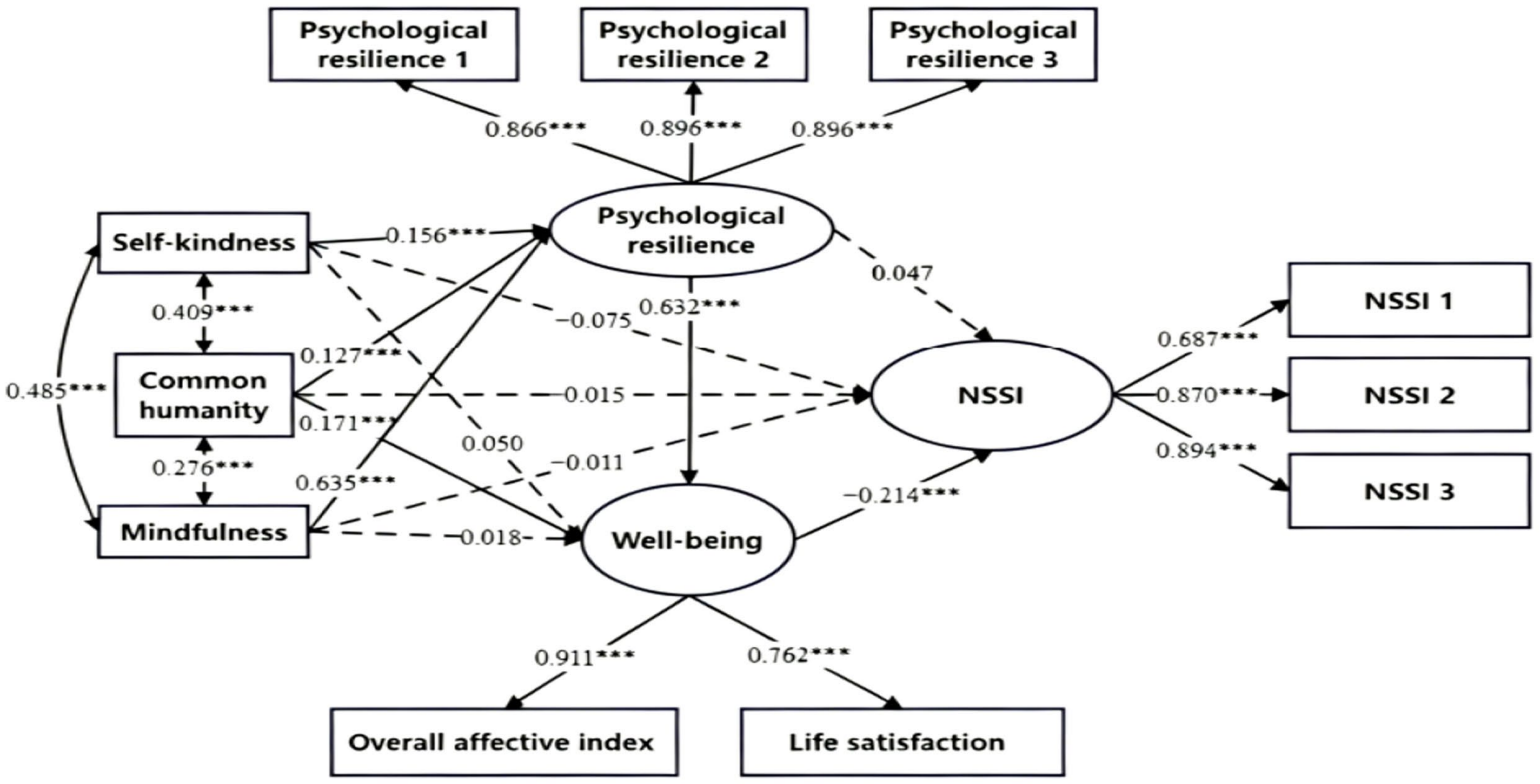
Diagram of the chain mediating effect model between components of self-compassion and NSSI. *p < 0.05, **p < 0.01, ***p < 0.001.

Psychological resilience was found to significantly and positively predict well-being (*β* = 0.632, *t* = 14.290, *p* < 0.001), whereas it exhibited no significant predictive impact on NSSI (*β* = 0.047, *t* = 0.871, *p* > 0.05). In contrast, well-being was able to significantly and negatively predict NSSI (*β* = −0.214, *t* = −5.006, *p* < 0.001). The standardized coefficients for all paths in the model are presented in Figure 3.

Bootstrap test with 5,000 resamples was conducted to examine the significance of the multiple mediating effects. As shown in Table 4, all components of self-compassion exerted significant predictive effects on NSSI through the chain mediating pathway from psychological resilience to well-being (*β* = −0.021, SE = 0.006, 95% CI [−0.035, −0.011]; *β* = −0.017, SE = 0.005, 95% CI [−0.029, −0.008]; *β* = −0.086, SE = 0.020, 95% CI [−0.128, −0.051]). In addition, common humanity also predicted NSSI through the mediating role of well-being (*β* = −0.037, SE = 0.009, 95% CI [−0.057, −0.020]). Further analyses revealed that the indirect effect of mindfulness on NSSI was stronger than those of self-kindness and common humanity (Wald test: *χ^2^*(1) = 11.266, *p* < 0.001; Wald test: *χ^2^*(1) = 6.286, *p* = 0.012).

**Table 4:** Bootstrap analysis for the significance test of mediating effects.

Mediating model pathway	Estimated effect	95% CI	
		Lower	Upper
Common humanity → well-being → NSSI	−0.037	−0.057	−0.020
Self-kindness → psychological resilience → well-being → NSSI	−0.021	−0.035	−0.011
Common humanity → psychological resilience → well-being → NSSI	−0.017	−0.029	−0.008
Mindfulness → psychological resilience → well-being → NSSI	−0.086	−0.128	−0.051

## 5 Discussion

Cultivating self-compassion is of great significance for promoting the development of college students’ physical and mental health. Accordingly, the present study recruited college students as participants, aiming to thoroughly examine the effects of self-compassion and its components on NSSI among college students, as well as the multiple mediating roles of psychological resilience and well-being. The findings demonstrated that strengthening college students’ ability to cope with difficulties and enhancing their internal positive psychological resources may contribute to decreased risk of NSSI behaviors. These findings provide new insights and implications for the scientific prevention and intervention of NSSI among college students, with important theoretical significance and practical value.

### 5.1 The relationship between self-compassion and NSSI

Consistent with findings from previous similar studies (Per et al., 2025), the present study revealed that self-compassion and its components were significantly negatively correlated with NSSI behaviors. That is, the higher the level of self-compassion among college students, the lower the likelihood of engaging in NSSI, which supported Hypothesis H1. As a protective factor for maintaining individual mental health, self-compassion may guide individuals to tend to adopt constructive strategies such as kindness, acceptance, and positive understanding during emotion regulation, while possibly decreasing the likelihood of employing maladaptive strategies such as avoidance, self-criticism, and emotional suppression (Hansen et al., 2021; McBride et al., 2022; Paucsik et al., 2023).

The optimization of emotion regulation styles not only facilitates the positive transformation and adaptive improvement of individuals’ internal psychological states but also enables them to respond rationally and adopt appropriate and effective strategies when facing adversities (Gamli et al., 2025; Mao et al., 2022; Meng et al., 2025). These results suggest that for students at high risk of NSSI, on the one hand, educational guidance and dynamic monitoring should be strengthened, and targeted interventions should be implemented to help them improve their self-compassion levels; on the other hand, relevant psychological training should be provided to teach self-compassion skills and guide them to build a positive psychological structure, thereby reducing the risk of NSSI.

### 5.2 The indirect mediating effect of psychological resilience

The present study found that psychological resilience did not mediate the relationship between overall self-compassion, its components, and NSSI among college students, and Hypothesis H2 was not supported. A possible reason for this result is that the direct risk trigger for individuals to engage in NSSI may lie in the imbalance of their internal emotional experience, especially the accumulation of negative emotional experiences (Brown et al., 2022; Poudel et al., 2022; Qu et al., 2023). When individuals experience internal emotional disharmony, the emergence of negative emotions such as anxiety and depression may exacerbate their risk of physical and psychological discomfort and further amplify their perception of distress. Such shifts in perception may correspondingly reduce individuals’ distress tolerance, potentially leading them to engage in self-injurious behaviors as a way to alleviate negative emotions (Calvete et al., 2022; Su et al., 2025). Although psychological resilience is a crucial protective factor against the risk of NSSI, as a relatively stable internal trait when individuals cope with stress, it is likely to exert indirect effects on individuals’ psychology and behavior only by regulating their internal psychological experiences.

### 5.3 The indirect mediating effect of well-being

The present study revealed that well-being functioned as a mediating role in the relationship between overall self-compassion, common humanity, and NSSI among college students, whereas no such mediating role was found between self-kindness, mindfulness, and NSSI. Thus, Hypothesis H3 was partially supported.

This result may be related to the unique characteristics of common humanity in the process of cognitive restructuring (Song et al., 2025; Yu, 2024). Common humanity is often associated with individuals’ general understanding and acceptance of their own experiences, making them more likely to recognize that suffering is a shared human experience. This pattern may help individuals transcend a one-sided and isolated perspective of their own distress, enabling a cognitive shift from a partial to an integrated framework, and may be associated with less rumination on negative cues and reduced amplification of negative emotional experiences.

In turn, it may provide individuals with a crucial sense of connectedness, which may also foster a more positive and tolerant orientation toward understanding and addressing their difficulties. In contrast, although self-kindness and mindfulness may offer immediate supportive attention, individuals may show insufficient transition from a state of mindful awareness to deep psychological adjustment in the absence of the connectedness and broad cognitive framework established by common humanity. This pattern may be linked to the less evident enhancement of well-being. Thus, common humanity may serve as a key component through which self-compassion relates to enhanced cognitive flexibility and higher levels of well-being.

### 5.4 The chain mediating roles of psychological resilience and well-being

The present study found that psychological resilience and well-being played a chain mediating role between overall self-compassion, its components and NSSI among college students, confirming Hypothesis H4. As a positive psychological quality integrating multiple cognitive and emotional coping styles, self-compassion may guide individuals to achieve emotion regulation through specific strategies including self-kindness, common humanity, and mindfulness when encountering difficulties. This regulatory process serves a dual functional role: on the one hand, it may reduce the intensity of negative emotional responses and help individuals build a harmonious and stable internal psychological environment (Mey et al., 2025); on the other hand, it may stimulate internal self-care and constructive positive forces, providing individuals with persistent resilient support when facing stress and setbacks (Zhao et al., 2025). These changes may be accompanied by increased well-being in individuals, and to some extent reduce the likelihood of NSSI resulting from emotional dysregulation.

Notably, although self-compassion and its components exerted no significant direct effects on NSSI, they may be indirectly associated with lower levels of NSSI through the mediating pathways of psychological resilience and well-being. Further analyses demonstrated that all indirect pathways included well-being. Therefore, psychological resilience and well-being may serve as important mediating factors in the relationship between self-compassion and individuals’ NSSI, and well-being may act as a crucial mediating factor between self-compassion and NSSI. In addition, the results further demonstrated that the indirect effect of mindfulness on NSSI was significantly stronger than those of the other pathways. A possible explanation is that mindful awareness may simultaneously integrate multiple regulatory functions across cognition, emotion, and behavior (He and Liu, 2023). Thus, its positive association with physical and mental health may be more prominent compared with other components of self-compassion. Furthermore, mindfulness may be associated with individuals responding to stress through active engagement rather than avoidance (Arslan and Asici, 2022), which may encourage them to deal with dilemmas more rationally, alleviate the adverse effects of stress, and be related to a lower likelihood of NSSI. Therefore, mindfulness may be a critical component linking self-compassion to lower levels of NSSI through specific mediating pathways. These findings suggest that future interventions targeting college students with NSSI should be oriented toward improving their internal emotional experiences. Cultivating individuals’ psychological resilience and well-being, with a particular emphasis on targeted strengthening mindfulness training, may effectively enhance the intervention effect of self-compassion on NSSI.

In summary, the study found that although overall self-compassion and its components showed no significant direct effects on NSSI among college students, they were associated with lower levels of NSSI through specific mediating pathways. Specifically, well-being played a significant independent mediating role between overall self-compassion as well as common humanity and NSSI, whereas psychological resilience showed no independent mediating role in the model. However, the direct effect of psychological resilience on well-being remained stable in the overall framework, and the indirect effect of mindfulness on lower levels of NSSI through specific mediating pathways was the most prominent. These results reflect the potential complex relationship between self-compassion and NSSI among college students, and may provide certain references for the development of intervention programs targeting college students’ NSSI.

### 5.5 Practical implications

The findings of this study offer important practical implications for mental health education in higher education and clinical psychological intervention. They provide several evidence-based references and practical pathways for the prevention and intervention of NSSI among college students, while also offering practical insights for mental health practitioners serving college students, including university mental health educators and professional psychotherapists.

First, both the mediation model and the chain mediation model highlight the potentially critical role of well-being in NSSI intervention. Mental health practitioners should emphasize the potential value of enhancing well-being in the prevention and intervention of NSSI among college students. Grounded in positive psychology theories and integrated with positive psychological intervention techniques, it is recommended to develop well-being-centered psychoeducational programs and implement them in mental health courses, group counseling, and individual psychotherapy. For instance, guiding students to identify and utilize their personal strengths, cultivate positive mindsets, and construct meaning in life can help them engage in positive intentional activities, thereby reducing the risk of NSSI engagement.

Second, the chain mediation model further demonstrates the potential positive role of psychological resilience in indirectly protecting against NSSI by enhancing well-being. In light of these results, targeted interventions focusing on psychological resilience are necessary in the prevention and intervention of NSSI among college students. This finding suggests that mental health practitioners should develop systematic intervention programs centered on psychological resilience to help college students gradually shift from passive stress coping to active adaptation and facilitate their psychological recovery and growth. For example, solution-focused reviews of “exceptional experiences,” role-playing scenarios targeting growth mindset, resilience-themed group counseling, and peer support groups can be adopted to help college students explore their potential internal resources for coping with adversities, thereby providing intrinsic support for reducing the risk of NSSI engagement.

Furthermore, the present findings also offer important implications for targeted interventions on self-compassion. Given the divergent pathways of the different components of self-compassion, mental health practitioners may implement tiered and differentiated intervention designs in practice. For students who engage in NSSI or are at risk of NSSI, intervention programs may appropriately emphasize the protective role of the mindfulness component by introducing training such as mindful breathing, emotional awareness, and mindfulness meditation, so as to improve their emotion regulation abilities and help them respond to stressful situations more rationally. This can be supplemented with guidance on self-acceptance to strengthen self-kindness and the perception of common humanity. For the general student population, self-compassion skills can be promoted through mental health courses or group counseling, with an emphasis on mindfulness training and common humanity strategies. They should be encouraged to share their common difficult experiences in group settings, with positive guidance, thereby enhancing their overall psychological resilience and well-being.

Finally, it should be noted that individuals with NSSI often present with some degree of cognitive impairment (Jin and Peng, 2026), whereas traditional self-compassion training relies to a certain extent on cognitive resources (Song et al., 2025). Therefore, in practical interventions, intervention formats may be optimized to reduce cognitive burden, and innovative efforts may be made to explore self-compassion regulation strategies that rely less on cognitive control and consume fewer cognitive resources, so as to address the limitations of existing approaches and improve their applicability and intervention effectiveness in real-life settings.

## 6 Limitations and future directions

Despite its theoretical and practical value, this study has several limitations that should be addressed in future research. First, the present study is limited by certain characteristics of the sample. The participants were Chinese college students from three universities in Zhenjiang, Nanchang, and Fuzhou, China. While geographic coverage was somewhat expanded, the overall scope remained relatively limited, which may not accurately reflect the true characteristics of the general population. Such sample homogeneity caused by geographic concentration may increase the risk of expectation effects and limit the generalizability of the findings beyond Chinese college students. Accordingly, future studies should optimize the sampling design in multiple ways: on the one hand, the sample coverage should be broadened to include participants from diverse cultural backgrounds, geographic regions, and university types to improve the generalizability and external validity of the findings; on the other hand, on the basis of enhancing sample diversity, future research can further focus on specific and more homogeneous groups for in-depth investigation, which will help strengthen the specificity and practical implications of the results.

Second, this study adopted a cross-sectional design. Although prior theoretical and empirical evidence provided a solid foundation for this study, the nature of this design makes it difficult to examine the temporal changes and causal relationships among variables. In addition, the scale used in the present study mainly assessed general self-compassion attitudes, lacking fine-grained evaluations of specific situations and emotional directedness. Given that self-compassion is characterized by dynamic fluctuations (Mey et al., 2023; Neff et al., 2021) and that college students are still in a period of physical and psychological development, future research should integrate experimental designs and longitudinal approaches to further examine the relationships among variables and reveal their dynamic processes and underlying mechanisms. For instance, future studies should design and implement intervention programs to systematically examine whether interventions targeting overall self-compassion and its distinct components lead to sustainable improvements in individuals’ physical and mental health, as well as test the mediating roles of psychological resilience and well-being in this process. Therefore, the findings of this study can only demonstrate correlational associations among variables rather than definitive causal relationships, highlighting the need for more comprehensive research methodologies in future investigations.

Third, this study has certain limitations in terms of variable control, primarily manifested in the lack of statistical control for demographic variables such as age, gender, and grade level. However, it should be noted that some methodological researchers have argued that while statistical control of extraneous variables is common in non-experimental research, the inclusion of control variables may not necessarily improve the reliability of the findings (Spector and Brannick, 2011). In correlational studies, merely removing shared variance between extraneous variables and antecedent variables through statistical methods may not equate to causal inference as in experimental research (Cao et al., 2020; Meehl, 1971). Additionally, due to the complex relationships among variables, statistically controlled results may sometimes appear ambiguous or even contradictory to the hypotheses (Becker, 2005; Breaugh, 2008). Nevertheless, the absence of control for the aforementioned demographic variables in this study may still introduce certain confounding effects, thereby introducing uncertainty into the precise estimation of the relationships among variables. Future research could, with a cautious approach, appropriately incorporate relevant control variables during the research design or statistical analysis stages to further enhance the robustness of the conclusions.

In addition, all data in the present study were obtained from college students’ self-reports. Although the results indicated no severe common method bias in the data, social desirability bias may still have influenced participants’ responses, thereby reducing the reliability of the findings to some extent. Accordingly, future research should adopt comprehensive data collection from multiple informants or use a multi-source reporting approach, such as integrating teacher ratings (e.g., daily behavioral observations), peer evaluations (e.g., companions reports), and parental assessments (e.g., family context behavioral reflections), to overcome the limitations of relying on a single source and further improve the reliability and stability of the results.

Finally, this study only examined the mediating roles of psychological resilience and well-being in the relationship between self-compassion and NSSI among college students, while the occurrence and development of NSSI may be associated with multiple factors. Notably, this study did not include key NSSI-related variables, such as depression, anxiety, impulsivity, and traumatic experiences (Jiao et al., 2022; Li et al., 2023), which may make it difficult to fully rule out their potential associations and confounding effects, and to some extent, limits the comprehensiveness of the current research model. Future research could, under a more integrated theoretical framework, attempt to further incorporate these key variables to explore their potential associations in the relationship between self-compassion and NSSI among college students, thereby more comprehensively revealing the relationship model in this population.

## 7 Conclusion

The present study found that self-compassion and its components were significantly negatively correlated with NSSI behaviors. Further analyses revealed that self-compassion was associated with lower levels of NSSI among college students not only through the independent mediating role of well-being, but also through the chain mediating pathway of psychological resilience and well-being. In addition, the present study also found that the indirect pathways through which the components of self-compassion were associated with NSSI might differ.

Specifically, common humanity was negatively associated with NSSI either through the independent mediating role of well-being or through the chain mediating role of psychological resilience and well-being. By contrast, self-kindness and mindfulness were negatively associated with NSSI through the chain mediation of psychological resilience and well-being. Furthermore, the indirect effect of mindfulness on NSSI was significantly stronger than those of the other components. These findings contribute to a deeper and more nuanced understanding of the complex relationship between self-compassion and NSSI, and also provide implications for designing effective prevention and intervention programs.

## Data Availability

For privacy reasons, the datasets analyzed in this study are not publicly available. However, they are available from the corresponding author, GH (1000005532@ujs.edu.cn), upon reasonable request.
